# Improving Personal Characterization of Meaningful Activity in Adults with Chronic Conditions Living in a Low-Income Housing Community

**DOI:** 10.3390/ijerph120911379

**Published:** 2015-09-11

**Authors:** Carrie A. Ciro, Patsy Smith

**Affiliations:** 1 Occupational Therapy Program, College of Allied Health, University of Oklahoma, 1200 N. Stonewall Avenue, Oklahoma City, OK 73117, USA; 2 College of Nursing, University of Oklahoma, 1100 N Stonewall Ave, Oklahoma City, OK 73117, USA; E-Mail: patsy-smith@ouhsc.edu

**Keywords:** meaningful activity, occupational therapy, public housing, mixed-methods, (feasibility) study, behavior change intervention, ageing

## Abstract

Purpose: To understand how adults living in a low-income, public housing community characterize meaningful activity (activity that gives life purpose) and if through short-term intervention, could overcome identified individual and environmental barriers to activity engagement. Methods: We used a mixed methods design where Phase 1 (qualitative) informed the development of Phase 2 (quantitative). Focus groups were conducted with residents of two low-income, public housing communities to understand their characterization of meaningful activity and health. From these results, we developed a theory-based group intervention for overcoming barriers to engagement in meaningful activity. Finally, we examined change in self-report scores from the Meaningful Activity Participation Assessment (MAPA) and the Engagement in Meaningful Activity Survey (EMAS). Results: Health literacy appeared to impact understanding of the questions in Phase 1. Activity availability, transportation, income and functional limitations were reported as barriers to meaningful activity. Phase 2 within group analysis revealed a significant difference in MAPA pre-post scores (*p* =0.007), but not EMAS (*p* =0.33). Discussion: Health literacy should be assessed and addressed in this population prior to intervention. After a group intervention, participants had a change in characterization of what is considered healthy, meaningful activity but reported fewer changes to how their activities aligned with their values.

## 1. Introduction

Ecological public health models emphasize that multiple determinants contribute to individual health including intra/interpersonal factors, environmental settings and societal contributions [1,2,3,4]. Some of these health determinants are modifiable, such as increasing physical activity, while some determinants are less amenable to change. For example, housing is an important environmental variable of health which may be difficult for an individual to modify due to limited resources. In particular, state or federally-subsidized low-income apartment units contribute to health in multiple ways. Stresses related to overcrowding, exposure to illness, financial stresses and lack of transportation have been reported [1,2]. People who live in public housing also tend to have higher rates of chronic conditions such as diabetes and high blood pressure and lower rates of physical activity due to environmental barriers (e.g., no sidewalks, unsafe walking environments), compared to similar residents not living in public housing [5]. On the other hand for some, low income housing communities provide a stable residence which reduces stress and provides opportunity for socialization [6]. Research which aims to improve health for residents of these environments, should take into account the unyielding positive or negative influences that this environment may play in overall health [4].

While the housing environment may be a difficult health determinant to influence, increasing engagement in meaningful activity is a modifiable health determinant and can improve health even when the environment is fixed [7,8,9]. The Model of Successful Aging suggests that engagement in meaningful activity is a key determinant of older adult enjoyment and adaptation to the aging process [10]. Meaningful activity can be thought of as activities that give one’s life purpose and fulfill a goal that is culturally or personally-relevant [11,12]. Engagement in meaningful activity is associated with improvements in sense of mastery, self-worth, quality of life, happiness and mental and physical health [9,13,14,15]. Each person values activities differently and as such, meaningful activities (occupations) vary widely across individuals, cultures and societies [14]. Some meaningful activities can be routine, such as bathing, grooming and dressing, and contribute to creating and maintaining one’s self-image. Other meaningful activities extend to caring for your home, caring for others (including pets), community participation and social/leisure engagement [11]. Adults with chronic conditions and particularly those living in low-income housing communities are at risk for not being able to engage in meaningful activities due to loss of function and limited resources [7,9].

The Well-Elderly studies comprise two large randomized controlled trials that examined the impact of occupational therapy on measures of mental and physical health in community-dwelling older adults [9,15]. The intervention, called Lifestyle Redesign^®^, was a series of individual and group sessions which focused on educating older adults about the power of meaningful activity and how to problem solve barriers to activity engagement. Findings demonstrated robust improvements in mental and physical health as well as delays in decline compared to comparator groups [9,15]. However, the Well-Elderly studies were funded, long term-interventions (6–9 months) completed on the West coast of the United States (U.S.) where perceptions of health may differ from participants in the southern U.S. Not known is the impact of a shortened, intervention in an area of the U.S. where characterization of healthy values/activities may differ and an understanding of health-related concepts, such as meaningful activity, health and wellness may be less understood [16].

Therefore, the purpose of this pilot study was to understand how older adults living in a low-income, residential community in the southern U.S. characterize meaningful activity and health and could overcome self-identified individual and environmental barriers to activity engagement through short-term intervention. Secondarily, we sought to understand what facilitators and barriers to engagement in meaningful activity exist within the infrastructure of the housing community. We developed questions that provided perspective on: (1) what the meaningful activities of the residents were and what obstacles prevented engagement; (2) how this environment supports meaningful activity or the barriers which may influence participation; (3) the extent to which healthy or unhealthy behaviors are recognized in others; and (4) the type of health information the residents desire to improve health and wellness. In our second study objective, we sought to examine the impact of a short-term group intervention (informed and created by findings from the first objective) on perception of and engagement in meaningful activity. We hypothesized that a seven week intervention with a focus on overcoming individual, contextual and social barriers would correspond with significant change in characterization of and engagement in meaningful activity. The original Well-Elderly studies were 6 and 9 months long which we were not able to implement due to lack of funding for this pilot project. We instead chose a brief intervention time frame which we hypothesized may be both effective and appealing to participants in this environment.

## 2. Experimental Section

### 2.1. Design

This project was an exploratory, sequential mixed methods design [17] where Phase 1, a qualitative (QUAL) focus group, informed the development of Phase 2, a quantitative (QUAN) quasi-experimental study examining the effect of an occupation-based health education group. A convenience sample was recruited for both phases. In Phase 2, measurements were taken at baseline and post-intervention. This study was approved by our university’s Institutional Review Board and the Oklahoma Housing Authority.

### 2.2. Participants

Our sample lived in low-income residential towers located near the campus of our university. Inclusion criteria: (1) residents of one of two residential communities aged ≥ 55; (2) Mini-Cog Screening Tool scores ≥ 2 [18]; (3) independently able to get to and from the classroom.

We employed a sequential, sampling method [19] from a convenience sample of residents living in the residential towers. We held two different recruitment sessions for each phase, but both were conducted in the same manner. First, tables were set-up in the library and lobbies of the two towers of interest. Fliers explaining both phases of the study were provided and research staff answered questions. A second table was set-up up for eligibility screening, the informed consent process and baseline evaluations. For those that had difficulty reading, staff read documents and/or questions to them. Participants of Phase 1were asked to participate in Phase II, but any anyone could also enroll in only one phase. No compensation was provided in either phase of the study.

### 2.3. QUAL Procedures and Measure

The first research objective was qualitative in nature, and thus we employed focus groups to understand resident perspectives. Focus groups rather than individual interviews were chosen to improve candor in this socially-marginalized groups and provide comfort for those speaking on subjects that may be considered private [17]. The focus groups were semi-structured with open-ended questions lead by the 2nd author. While we had a structured question path, ideas and comments were expanded upon or explained as needed. One focus group was held in the library of one of the towers. Three research staff were employed to act as the interviewer (Patsy Smith), the recorder (Carrie Ciro) and a research student who assisted with set-up. A recorder was used to tape the interviews which lasted one hour. Participants in Phase 1 were asked if they would be willing to participate in Phase II and consenting volunteers provided contact information. The structured questions were:

Environment

1. How is *activity* encouraged for people that live here?
2. Some of you do not participate in these activities just discussed. Why are they less attractive for you?

Meaningful Activities (Occupations)

1. Tell me about the meaningful activities that you do every day.
2. What problems or obstacles would have to be removed for you to perform the way you desire?

Health

1. What do healthy people look like and act like in the center? What do unhealthy people look like and act like in the center?
2. What would you like to learn about your health and wellness?

### 2.4. QUAN Procedures and Measures

Our second research objective was quantitative in nature, and thus we used appropriate methodology and outcome tools to measure change. Four months after we completed the focus group, we recruited for and began the second phase of the study. We gathered descriptive data at baseline and outcome data at baseline and post-intervention.

A *sociodemographic profile* was used to gather general sociodemographic data on age, gender, race/ethnicity, education, living situation, self-reported diagnoses (e.g., high blood pressure, diabetes, depression, mental illness) and general functional level (e.g., assistance with walking and activities of daily living).

The *Center for Epidemiological Study-Depression (CES-D)* is a self-report, 20-item scale designed to measure depressive symptoms in community-dwelling and hospitalized older adults [20,21]. The scale asks how often a person has had a depressive symptom over the past week using a 4-point ordinal scale: rarely or none of the time (less than 1 day); some or a little of the time (1–2 days); occasionally or a moderate amount of the time (3–4 days); most or all of the time (5–7 days). The CES-D measure has a potential range of 0–60, where a score of ≥16 is categorized as high depressive symptoms. In older adults, the internal consistency is excellent (Cronbach’s α = 0.85–0.91) [20].

The *Personal Resource Questionnaire 2000* is a self-administered 15-item instrument designed to measure perceived social support. Questions cover a range of social support constructs and includes items such as, “There is someone I feel close to that makes me feel secure.” Individuals respond to questions using a 7-point Likert scale where 1 = strongly disagree, 2 = disagree, 3 = somewhat disagree, 4 = neutral, 5 = somewhat agree, 6 = agree and 7 = strongly agree. Scores range from 15–105. Lower scores indicate lesser perceived social support, and higher scores indicate greater perceived social support. There is no identified “cut” score at which lower scores are differentiated from higher scores. A systematic review yielded excellent internal consistency (Cronbach’s alphas ranged from 0.87 to 0.93) [22].

The *Meaningful Activity Participation Assessment (MAPA*-global version) is an 8-item self-report instrument designed to measure the extent of understanding that meaningful activity underlies health. An example question is, “Overall, how much do you think your activities contribute to your mental health?” Questions are answered on a 1–7 Likert scale where “1” indicates *not at all* and “5” indicates *a great deal.* Scores range from 8 to 56 where a higher score indicates a greater understanding of activity and health. In a general population, internal consistency (α =0.85) and test-retest reliability (*r* = 0.84) are good [23].

The *Engagement in Meaningful Activity Scale* (EMAS) is a 12-item self-report instrument to measure the extent that one’s activities align with values, have value within one’s social structure and demonstrate the person’s mastery of meaningful activity. An example question is, “The activities I do are valued by other people.” Questions are answered on a 1–5 Likert scale where “1” indicates *(never)* and “5” indicates *(always).* Score ranges from 12 to 60 where higher scores indicate high engagement in meaningful activities that align with your personal values. Factor analysis of the primary components of the EMAS suggest a two-component structure, Social Experiential and Personal-Competence [24]. In a general population, the internal consistency (α = 0.89) and test-retest reliability (*r* = 0.56) are moderate-good [24].

### 2.5. QUAL Data Analysis

Audio recordings from the focus group were transcribed verbatim. Open coding, the process of looking for quotes or themes [25], was conducted by the first author (Carrie Ciro) and a graduate assistant trained in coding assisted with the group. First, separate analyses were conducted and then agreement on coding was achieved collaboratively. Because the participants in the focus group tended to simply agree verbally or non-verbally with 1–2 people that were answering, we did not collect multiple answers on questions. Therefore, we provided statements that appeared to be consensus statements for the group. We were not able to triangulate findings with the representatives from the focus group.

### 2.6. QUAN Data Analysis

Means ± standard deviations (SD) and ranges were reported for continuous variables and proportions for categorical variables. Wilcoxon signed rank tests were used to examine within groups differences for pre and post scores in our outcome measures with ordinal data. In *post-hoc* analysis, individual item change scores were examined using signed rank tests and significance levels were adjusted using Bonferroni corrections. Statistical significance for all tests was set at *p* < 0.05 and data were analyzed using Statistical Analysis System (Version 9.2, SAS Institute, Cary, NC, USA).

### 2.7. Data Integration

As this was a mixed methods design, we used the data from Phase 1 (QUAL) to inform the development (content and sequence) of the occupation-based education groups in Phase 2 (QUAN). This methodology allowed the research team to “tailor” the group intervention based on focus group feedback.

## 3. Results

In the exploratory, sequential design, the qualitative data informs the quantitative study. Therefore, the QUAL results are provided here prior to the QUAN.

### 3.1. QUAL Results

Focus groups were organized through three question paths: (1) the environment; (2) meaningful activity; and 3) the resident’s understanding of health and wellness. In the first set of questions related to the environment, residents were asked *how activity was encouraged for people that lived in the residential centers?* Largely, activities are “available” to those that seek them such as areas for dominoes, pool tables, and picnic areas with grills. Outside groups come in to provide some of the activities such as bible study and health education groups. In one tower, an adult day center is available with daily activities but the enthusiasm of the group leader largely dictates interest by the residents. A computer room is available but residents report needing training. Fliers for tower activities are posted in common areas in one tower but not the other leaving some complaining that they didn’t know when activities were offered. In a second question related to environment, residents were asked *why are those activities less attractive for you?* The majority noted that there were not enough activities and those available did not align with their interests. For example, one male resident wanted more outdoor activities such as shuffleboard and horseshoes, instead of indoor craft activities. Other residents wanted more bible study and in-door exercise. A second reason people did not attend was that the activities were not adapted to their special needs such as low vision. One woman said, “I cannot see the thread through the needle…so I just gave up.” A third barrier to activity was lack of transportation. All were enthusiastically grateful for the housing community buses that took them to day appointments, grocery stores and special outings (e.g., zoo, museums, lunch). However, the buses were not available every day of the week and not for evening and weekend activities such as going to church, family get-togethers or evening movies. Taxis were “too expensive for my blood” and other transportation sources were not reliable. One woman noted that a local transportation service frequently told her, “We can get you there but can’t get you back.” To which she responded, “How is a woman in a wheelchair going to get her way back if someone doesn’t take her?”

In the second set of questions, we asked about meaningful activities. When this question was asked the first time, it was framed to ask, “Tell me about the meaningful activities you do every day.” The respondents began to describe their daily activities. To help focus responses, we defined meaningful activity as the activities that are so important, that if you could not do them, it would affect your happiness. However, the responses were still not rich and residents did not appear to understand what was meant by “meaningful activities” so the wording was changed to *what do you do during the day?* Residents reported a large variety of activities, many of which involved socialization or watching other people in the towers. For example, one woman said she liked to sit by the elevator because, “you can see everybody coming out and coming in.” Others noted afternoons playing dominoes, playing pool and visiting with others. Several residents lived by the routines of weekly schedules, such as “I do my laundry on Wednesday” and another noted, “I do bible study Tues and Thursday night when the church bus picks me up.” Cooking was an activity that several mentioned and enjoyed doing with others. In a second question related to meaningful activities, we asked *what problems or obstacles would have to be removed for you to perform the way you desire?* Most reported that they were able to do what they wanted to do but were restricted primarily by income and having transportation to go where they want to do. All have used the bus offered by the center to attend outside events such as going to the zoo, art museums, or doctor’s appointments. Several have had bad experiences using local, income-based transportation services that were often not on time. One woman with an amputation just stopped doing what she could not do, adding, “You need to learn to be an adult and face up to it.”

In the last set of questions, the focus was on health and wellness. We wanted to know *what residents thought healthy people looked like* followed by a question about *what unhealthy people look like?* The first question yielded few results. Residents reported that no one that lived in the towers was healthy because they all had problems that might be physical or mental. To probe deeper, we asked *what kind of healthy behaviors do you see people display in the towers?* Two residents reported “people helping other people,” followed by a supportive comment rooted in faith and spirituality, “you reap what you sow, you sow good seeds, you reap good seeds!” In the follow-up question about what unhealthy people looked like, they tended to report medical conditions such as diabetes, stroke, dementia, blindness, oxygen use, mental illness and substance abuse. Other residents supported this way of thinking by describing people using wheelchairs and walkers. In our final question, we asked *what the residents would like to learn about their own health and wellness?* Each person noted their own medical conditions, such as diabetes, high blood pressure, asthma and chronic obstructive pulmonary disease. When asked specifically, the residents said they preferred information about “how to manage” their diseases over disease-specific information.

### 3.2. QUAN Intervention

The theoretical foundation for the intervention was Rowe and Kahn’s Model of Successful Aging [26] in whichsuccessful aging is accomplished by avoiding disease and disability, active engagement in meaningful activity, and maximizing physical and cognitive abilities *Active engagement in meaningful activity* refers to remaining productive in activities that hold personal value. *Avoiding disease and disability* refers to reducing the risk of disease through lifestyle changes informed by awareness of genetic and environmental predictors of health. Finally, one *maximizes physical and cognitive abilities* by engaging in activities that improve or maintain skills such as mobility, and short term memory [26]. The model has been modified to include *positive spirituality* given the evidence that engaging in formal or informal rituals/activities of religion or spirituality supports health in older adults [21].

Through this theoretical lens and informed by the analysis of focus group data, seven, one-hour groups were developed.  Content addressed the definition and power of meaningful activity in influencing physical and mental health, exploration of activities that address physical, cognitive and mental health, and solutions for overcoming environmental (social, physical and monetary) barriers which prevent participation in meaningful activity. The groups were called Adults Reaching for Occupation, Understanding, Strength and Engagement (AROUSE). AROUSE group content was modeled after the structure and content of Lifestyle Redesign^®^, the intervention groups provided by the Well Elderly studies, but modified for this audience due to the outcomes of our focus group, time, and funding restraints [8]. Emphasis was also placed on how meaningful activity, hence occupation, positively influences reported disease processes such as diabetes and high blood pressure.

The researchers lead a weekly, one-hour group in the activity room of each tower. Participants wore name tags to the first two groups until names were learned. Each group was prompted to create a name for their group to create cohesion within group members. Active and collaborative teaching models were used to engage individuals and spark conversation amongst group members. Educational materials built on the content from the last group, yet content was also designed to stand alone for those that missed any sessions. Table 1 provides information on the developmental and practical structure of the AROUSE groups. To aide in comparing this study to previous and future behavioral change research, we added Behavior Change Techniques [27] as a final column in Table 1, to describe the specific techniques employed during learning activities. Behavior Change Techniques are considered any process that may change psychological determinants, for example, self-efficacy and motivation [28].

### 3.3. QUAN Results

Table 2 notes the sociodemographic characteristics of both groups. Focus group members were primarily African American, lived alone and the majority had a high school or higher educational level. All focus group members were asked to participate in Phase II and half attended. The intervention group was primarily females who were either single or divorced with high school or higher education. All lived alone and self-reported some type of chronic physical or mental illness. The group means suggested high depressive symptoms and perceptions of moderate social support. No drop-out occurred within Phase 2 and the average attendance rate for seven group meetings was 91%.

**Table 1:** Adults reaching for occupation (meaningful activity), Understanding, Strength and Engagement (AROUSE) group structure.

#	Session Topic	Group Objectives	Model of Aging Theoretical Concepts *	Behavioral Change Techniques Employed
1	Understanding health and meaningful activity	1. Define health and meaningful activity. 2. Brainstorm activities that each considers healthy. Introduce idea of meaningful activities such as such as cooking with the family as a healthy activity. 3. Discuss and sort the activities by how each might contribute to improved performance in 3 areas of health: (1) physical. (2) cognitive. (3) mental 4. Reflect on how healthy activities can be chosen and prioritized to meet health goals for improving HBP and DM.	A, B, C	Tailored message, problem solving/ planning, prompts, cues, persuasive argument, health consequences.
2	Exploring your meaningful activity	1. Devise a schedule of their current daily activities for one week, including self-care, exercise, leisure and socialization. 2. Categorize their activities by emphasis on physical, cognitive or mental health if applicable. 3. Identify strengths and gaps in activities and set goals for weak areas.	B, C	Self-monitoring of behavior, social support, problem solving/planning, discrepancy between current and desired behavior, goal setting behavior.
3	How to overcome barriers to meaningful activities	1. Brainstorm a variety of barriers that interfere with meaningful activity engagement. 2. Problem-solve creative ways to overcome barriers including community resources. 3. Reflect on how they will use these suggestions to overcome barriers to the activities they would like to engage in.	B, C	Self-monitoring of behavior, restructuring of physical environment, social support, problem/solving and planning, persuasive argument, prompts, cues, discrepancy between current and desired behavior, tailored message.
4	Meaningful activity that promotes physical activity	1. Consider how physical exercise positively affects many healthy variables, such as lowering risk of death, reducing depression and improving sleep. 2. Understand differences in the 3 types of physical exercise recommended for older adults, cardiovascular, resistance training and balance training, as well as the dosage for improving health. 3. Practice using a pedometer (or for those in a wheelchair, an arm bike), elastic exercise bands and balance exercise under the supervision of the group leaders. (Elastic exercise bands issued to each participant for home use). 4. Reflect on how physical exercise may contribute to improving health in people with HBP/DM.	A, B, C	Health consequences and benefits, self-monitoring of behavior, instruction on how to perform behavior, behavioral practice, behavioral demonstration, goal setting, self-monitoring of progress, feedback on behavior, tailored message.
5	Meaningful activity that promotes cognitive health	1. Consider how mental exercise positively affects many health variables, such as lowering risk of dementia and maintaining independence in daily activity. 2. Understand how activities such as exercise, sleep, and socialization improve cognition; provide current evidence on dosage for effectiveness. 3. Brainstorm activities that require “thinking” (e.g. playing cards) and then identify the activities that they currently do that require thinking. 4. Develop a weekly schedule that includes physical, mental and mood-improving activities. 5. Reflect on how mental exercise may contribute to improving health in people with HBP/DM.	A, B, C	Health consequences and benefits, self-monitoring of behavior, instruction on how to perform behavior, behavioral practice, behavioral demonstration, goal setting, self-monitoring of progress, feedback on behavior, tailored message.
6	Meaningful activity that promotes mental health	1. Complete a social and leisure inventory of activities they engage in now or in the past. 2. Discuss within the group, how social and leisure activities improve health (e.g., prevent illness, improve mood and life satisfaction); provide appropriate dosage for effectiveness. 3. Brainstorm familial and community resources. 4. Engage in “play” with group members by trying a new card game and a new dice game while enjoying sugar-free snacks. 5. Reflect on how social and leisure activity may help HBP/DM and reinforce the need to put them in their written schedules devised last week.	A, B, C	Health consequences and benefits, self-monitoring of behavior, instruction on how to perform behavior, behavioral practice, behavioral demonstration, goal setting, self-monitoring of progress, feedback on behavior, tailored message.
7	Graduation and wrap-up group	1. Engage in a group overview, including “take home messages” from each group. 2. Review their very first daily schedule and compare to the last so that they can see the progress made in healthy activity selection. 3. Complete post-assessments. 4. Receive validation for their work and participation by receiving a formal certificate of training through a “graduation” ceremony.	A, B, C	Tailored message, non-specific encouragement, persuasive argument, review behavior goals.

* Model of Theoretical Aging Concepts: A = Avoiding disease and disability; B = Active engagement in meaningful activity; C = High cognitive and physical function.

**Table 2:** Description of sample for both qualitative (QUAL) and quantitative (QUAN) phases.

Demographics	QUAL Sample (*n* = 6)	QUAN Sample (*n* = 11)
Age Mean (SD:range)	62.5(4.37:57–68)	65.3 (8.86: 55–81)
Race	Black 6/6, Caucasian 0/6	Black 6/11, Caucasian 5/11
Gender	Male 3/6, Female 3/6	Male 4/11, Female 7/11
Marital Status	Single 3/6, Married 1/6, Divorced 2/6	Single 5/1l, Married 1/11, Divorced 5/11
Lives alone	5/6	11/11
Education	Below HS: 2/6, HS Graduate: 3/6, Post HS: 1/6	Below HS: 2/11, HS Graduate: 4/11, Post HS : 5/11
Health insurance	No: 2/6 Yes: 4/6, Not sure: 0/6	No: 1/11, Yes: 10/11, Not sure: 1/11
+ Hypertension	3/6	8/11
+ Diabetes	2/6	4/11
+ Depression	1/6	6/11
+ Mental illness	1/6	6/11
Assistance with walking	None: 4/6, WC/ scooter: 2/6, Walker: 0/6	None: 6/11, WC/scooter: 2/11, Walker: 3/11
Assistance to use bathroom	Trouble getting to restroom in time: 3/6	None: 9/11, Yes: 2/11
CESD Mean (SD; range)	NA	40 (13.70; 26–61)
PRQ 2000 Median (25th, 75th quartiles)	NA	83 (61,103)

HS = High School; CESD = Center for Epidemiological Study-Depression; PRQ 2000 = Personal Resource Questionnaire; WC = wheelchair

The MAPA was used to assess participant ability to understand the extent to which meaningful activity underlies health. The post-group MAPA score mean (*M* = 46.6; *SD* = 1.7; Range: 39–56) was significantly higher than the pre-group MAPA mean (*M* = 35.6; *SD* = 2.5; Range: 20–43) (*p* = 0.008). In a secondary analysis looking at individual MAPA item change scores, the items with significant change were, “How satisfied are you with your activities?” (*p* = 0.002), and “What change has there been in your overall activity level from six months ago to today?” (*p* = 0.004). Of note, there was a trend for significant change in the question, “Over the past 6 months, have your daily round of activities changed in terms of how meaningful or personally fulfilling they are for you?”(*p* = 0.08).

The EMAS was used to assess the extent that participant activities align with values, have value within the social structure and demonstrate the person’s mastery of meaningful activity. The post-group EMAS score mean (*M* = 51.1; *SD* = 2.1; Range: 41–60) was not significantly higher than the pre-group mean (*M* = 47.3; *SD* = 2.5; Range: 34–60). No individual EMAS items were significantly different across time.

## 4. Discussion

In the first phase of our project, residents reported that their housing community provided social and leisure activities but that the activities were not personally meaningful for the majority in this small sample. Also, limitations in individual function, such as vision, served as a barrier for activities provided by the housing community. Meaningful activities mentioned most often by the residents were social/leisure and cooking-tasks. Barriers to performing meaningful activities were primarily limited income and lack of transportation. “Health” was perceived as something no one in the center had as health was defined as lack of any physical or mental issue. The only healthy behaviors they identified seeing in one another was other people helping each other. Participants reported needing health education that focused on disease management. In the second phase of our project, we found that short-term group intervention with content tailored to this sample improved perception of meaningful activity but did not reach statistical significance in changing engagement in meaningful activity that aligned with their values and the values of their social systems.

### 4.1. Health Literacy and Focus Groups.

Focus groups were used to gain an understanding of resident perspectives on a number of topics. Residents provided limited responses to questions and displayed a tendency to shake heads in approval to comments of others *versus* providing one’s own opinion. Focus group questions were pre-rated at grade level of 5.4, yet respondents had a difficult time answering the questions directly [29]. One explanation for this could be reduced health literacy. Health literacy can be defined as how well one uses health-related information to describe, respond to and understand health needs [16]. African Americans and older adults, particularly those with chronic conditions are found to have lower health literacy levels which impact understanding medical jargon [16,30]. Our questions included words such as “health”, “unhealthy” “meaningful activities”, and “wellness” all of which could be arguably considered health-related terms. Future research would benefit from including assessment of health literacy prior to the study and as needed, strategies for improving health literacy, such as collaborating to define health concepts prior to preliminary interviews and interventions [31].

### 4.2. Environmental Barriers to Participation in Meaningful Activity

Despite difficulties with focus group participants answering questions, information was gathered which may be of use in similar settings. These residents noted that social/leisure activities were offered, but that the activities largely were not in alignment with their personal interests. In an environment with limited resources, it would be difficult to have activities that met all of the resident’s interests, a common issue in other settings [32]. However, this does not preclude community leaders from attempting to assess the scope of resident interest to examine feasibility of future inclusion [33]. A second environmental barrier was lack of awareness of what activities are currently available within the housing community. One tower posted a schedule, one did not. People with visual problems reported inability to read the small print on monthly calendars. Providing a daily or weekly schedule may reduce the visual overload associated with monthly calendars and enlarging the print on calendars for people with restricted vision may improve their participation [34]. A third environmental barrier discussed was transportation. The residents were grateful for the transportation (bus) that was available to them to go on outings (museum, zoo), trips to grocery stores and when available to doctor’s offices. However, they described needing transportation, 7 days/week, including limited night hours, to be with family, attend church or go out in the evenings. Public transportation was identified as a common problem for older adults who can no longer drive [35]. Taxis were found unaffordable and bus systems were too complex to negotiate Intervention focused on reading maps, planning routes and the actual use of public transportation would be beneficial for this group [9]. Finally, residents who participated in the adult day program reported that some activities offered were difficult to do because of functional limitations. Here an occupational therapy professional could be helpful in consulting on how to modify activities for individual residents [36]. In community settings where budgets are limited, occupational therapy educational programs may be of service in exchange for the opportunity to provide students with service learning opportunities.

### 4.3. How Understanding of Meaningful Activity, Health and Interconnectedness of Both Constructs Framed the Intervention Groups

Within our sample, there was decreased awareness of what is considered meaningful activity and little understanding of the contribution of meaningful activity to overall health. Clark *et al.* hypothesized that an older adult’s ability to abstract meaning from daily tasks is key to successful aging and an important role for occupational therapists [9]. The overarching goal of the AROUSE groups became to educate participants that meaning is found in everyday activities followed by collective brainstorming about how to overcome barriers they reported in order to stay engaged in those meaningful activities. Our participants created strategies such as: (1) ride sharing to movies to decrease costs; (2) “accountability” buddies for exercise; (3) gathering a group to petition for shared gardening space; and (4) open dialogues with friends and family about what their needs are. Collaboratively, the participants solved problems not only for their own barriers, but for all of those within the group. Other researchers have noted the benefits of pairing older adults together to solve interpersonal problems which supported our learning techniques [37]. Secondly, participants identified with a restricted view of health where “health” is considered the absence of any health issues. Indeed, good health is highly correlated with successful aging in older adults [38]. However, research also shows that for some, successful aging may require a reconceptualization of health and restructuring of activity goals, a process called compensation. Because of this finding, AROUSE group objectives highlighted health as a continuum state with periods of acuity and chronicity to reframe their concept of health as either good or bad. Furthermore, it was emphasized that while one dimension of health may be diminishing (e.g., physical health), other dimensions of health may be stable or strengthening (e.g., mental health) which provided focus for strengths in the reframing of health. Finally, the concepts of meaningful activity and health were tethered in a number of ways. For every meaningful activity discussed, participants specifically considered the impact on physical, cognitive and mental health. By having participants think about how muscles feel after standing in the kitchen to cook and how you emotionally feel when playing cards with your friend, more difficult connections were prompted. These learning strategies were modeled after Lifestyle Redesign®, the successful group intervention within the Well Elderly studies [7,9,39].

### 4.4. Perception of and Engagement in Personally-Meaningful Activity

After the short-term intervention, residents of this housing community appeared to improve in their awareness of meaningful activity as demonstrated by a significant difference in MAPA scores. However, there was not a significant change in how their activities aligned with values or how their activities were valued within his/her social structure as measured by the EMAS. These results seem to indicate that the types of activities did not change significantly, but participant assessment of the value of these activities and the understanding of the contribution of these activities to health did change. Ultimately, this may be an important outcome for people with limited resources for trying new activities. By understanding the importance of meaningful activity to health, these participants may have been willing to set goals which lead to the behavioral change of understanding and eventually, engaging in in valued activity [10].

### 4.5. Delivery Models for Future Interventions and Research

The AROUSE group intervention has potential application in a wide variety of settings that serve adults and older adults yet needs more research. When working with community-dwelling adults, the AROUSE group intervention may be conducted in community health centers, adult aging centers, adult day centers or outpatient clinics in the same manner delivered in the study. Also, the AROUSE group intervention may be useful for adults in inpatient environments such as behavioral health units, rehabilitation centers, and hospitals settings with a more frequent dosage such as 3–4 times week given the brevity of inpatient stays [40,41]. Individual delivery of the material is feasible in environments where groups are not possible, yet the benefits of group interaction for this intervention may be an important active ingredient. Randomized-controlled trials which emphasize recruitment strategies in these special populations, appropriate attention-control groups and the length of intervention needed for maximal efficiency and effectiveness are needed.

### 4.6. Limitations

Our findings must be interpreted through the limitations of the methodology. First, the qualitative methods were problematic in that the sample had lower health literacy than anticipated. Therefore, the use of focus groups may have been a limiting choice for this sample since they had difficulty responding to the questions. Also, despite prompting, the focus group participants tended to agree with one another through non-verbal communication rather than elaborate on personal opinions. This forced us to record qualitative responses descriptively without analyzing them collectively which further increases the risk for bias and reduces external validity. Second, we chose a quasi-experimental design for this pilot study and used convenience sampling to recruit participants. The sampling method introduces bias and the design limits our ability to make inferences. Furthermore, the sample was small due to difficulty recruiting in this environment. However, we were able to recruit our target audience of older adults with chronic conditions. Small sample size results can lead to larger effect sizes than would be seen in larger studies [42]. Therefore the significance values must be interpreted cautiously.

## 5. Conclusions

In our sample of residents in low-income housing, we found health literacy barriers which prevented a complete understanding of health, wellness and meaningful activity. Using the data we could obtain, a short-term intervention group was created and delivered on-site which resulted in improved personal characterization of meaningful activity and its connection to health, despite no significant change in how their activities aligned with values in the environment in which they lived.
